# Validation of the general self-efficacy scale in french students for the prevention of student health

**DOI:** 10.1192/j.eurpsy.2021.1226

**Published:** 2021-08-13

**Authors:** D. Saleh, L. Romo, S. Julien Sweerts

**Affiliations:** 1 Counseling Psychology, Tishreen University, Latakia, Syria; 2 Ea 4430 Clipsyd, Department Of Psychology, Université Paris Nanterre, Nanterre, France; 3 C2s Ea 6291, France, Université de Reims Champagne Ardenne, Reims, France

**Keywords:** measure, General Self-Efficacy, Prevention of mental health, stress

## Abstract

**Introduction:**

The perceived self-efficacy, framed by Bandura, is one of the most important concepts within Cognitive Social (Villegas Barahona et al., 2018). General self-efficacy is defined, as the global confidence a person has in order to perform tasks successfully (Stanley & Murphy, 1997). The perception of stress may be more for people with lower level of self-efficacy (Shilpa & Prasad, 2017).

**Objectives:**

Students often suffer from stress (Saleh et al., 2019) and student health intervention and prevention programs must therefore act on this variable. The French version the General Self-Efficacy Scale could be an element for the validation of these programs.

**Methods:**

955 French students aged 17 to 67 (M = 22.22; SD = 5.1) participated to the study. We performed an Exploratory Factor Analysis (EFA) to determine the most appropriate factor structure then a Confirmatory Factor Analysis (CFA).

**Results:**

Kaiser’s criterion pointed towards a one-factor model while Cattel’s criterion pointed towards a two-factor solution. Both models have been tested and the two-factor model seemed to be better. Indices showed an excellent fit between the model and the data (CFI = 0.97, TLI = 0.96, SRMR = 0.035).

**Conclusions:**

We have tested two models and one of them, the two-factor model, presented better psychometric qualities. However, the one-factor result is also satisfactory and it will be discussed in the communication.

